# Abnormal Olfaction in Parkinson's Disease Is Related to Faster Disease Progression

**DOI:** 10.1155/2015/976589

**Published:** 2015-06-02

**Authors:** Sara Cavaco, Alexandra Gonçalves, Alexandre Mendes, Nuno Vila-Chã, Inês Moreira, Joana Fernandes, Joana Damásio, Armando Teixeira-Pinto, António Bastos Lima

**Affiliations:** ^1^Serviço de Neurologia, Centro Hospitalar do Porto, 4099 Porto, Portugal; ^2^Unidade Multidisciplinar de Investigação Biomédica, Instituto de Ciências Biomédicas Abel Salazar, Universidade do Porto, 4050 Porto, Portugal; ^3^Faculdade de Medicina, Universidade do Porto, 4200 Porto, Portugal; ^4^CINTESIS, Faculdade de Medicina, Universidade do Porto, 4200 Porto, Portugal; ^5^Screening and Test Evaluation Program, Sydney School of Public Health, The University of Sydney, NSW 2006, Australia

## Abstract

*Introduction*. A possible association between olfactory dysfunction and Parkinson's disease (PD) severity has been a topic of contention for the past 40 years. Conflicting reports may be partially explained by procedural differences in olfactory assessment and motor symptom evaluation. *Methods*. One hundred and sixty-six nondemented PD patients performed the Brief-Smell Identification Test and test scores below the estimated 20th percentile as a function of sex, age, and education (i.e., 80% specificity) were considered demographically abnormal. Patients underwent motor examination after 12 h without antiparkinsonian medication. *Results*. Eighty-two percent of PD patients had abnormal olfaction. Abnormal performance on the Brief-Smell Identification Test was associated with higher disease severity (i.e., Hoehn and Yahr, Unified Parkinson's Disease Rating Scale-III, Freezing of Gait questionnaire, and levodopa equivalent dose), even when disease duration was taken into account. *Conclusions*. Abnormal olfaction in PD is associated with increased severity and faster disease progression.

## 1. Introduction

Olfactory dysfunction is a good predictor of future decline in cognitive and motor functions, including development of parkinsonian signs, in community dwelling older normal adults [1]. Hyposmia is one of the earliest manifestations of certain neurodegenerative diseases of the CNS, such as Parkinson's disease (PD). The impaired sense of smell can even precede clinically detectable motor signs by several years [2]. Olfactory deficits have been found in 70% to 90% of PD patients [3–8]. The literature has provided evidence that patients with more pronounced olfactory loss are at increased risk of developing dementia [9] and other neuropsychiatric complications [10] associated with PD.

Hyposmia in PD has been thought to be largely independent of disease duration, severity of motor symptoms, and current medication [3–5, 11–20]. However, small associations with disease duration and/or disease severity have been reported [21–26]. These inconsistent findings may be related to procedural differences in measuring olfactory dysfunction (e.g., use of different assessment instruments, interpretation of olfactory performance irrespective of demographic confounding factors). Most studies on the topic lack information regarding motor symptom assessment conditions of treated patients (i.e., “on” versus “off” medication conditions) or mix nonmedicated patients with patients assessed in “on” medication state.

The main goals of this study were to detect olfactory impairment beyond demographic effects in a cohort of patients with PD and to explore the association between abnormal olfaction and PD patients' motor characteristics.

## 2. Methods

### 2.1. Participants

#### 2.1.1. Normative Sample Group

The normative sample was composed of 388 healthy participants (70.1% women; 14.6% had current smoking habits; and 6.9% had past smoking habits) between 18 and 94 years of age (mean = 53.1; sd = 18.4) and between 3 and 21 years of education (mean = 9.9; sd = 5.3) living in the northern region of Portugal.

#### 2.1.2. Parkinson's Disease Group

One hundred and sixty-six nondemented patients with PD diagnosis (according to the United Kingdom Brain Bank criteria) were recruited consecutively from Centro Hospitalar do Porto's movement disorders outpatient clinic (Table 1). The inclusion criteria were ≥3 years of education and normal cognitive functioning on the Dementia Rating Scale-2 (DRS-2). Only patients with DRS-2 total score adjusted for age and education above the 5th percentile (i.e., estimated specificity of 95%), according to the national norms [27], were included in the study. All participants were also able to describe verbally their medication and its time schedule (i.e., the pill questionnaire test).

#### 2.1.3. Ethics

All participants provided their informed written consent to participate in this study, in accordance with the Declaration of Helsinki. The local ethical committee approved the study.

### 2.2. Procedures

#### 2.2.1. Assessment Protocol

Disease duration (in years) was calculated from the onset of subjective motor symptoms. The current antiparkinsonian medication was converted to levodopa equivalent dose [28]. Neurologists specialized in movement disorders assessed PD patients' motor symptoms after 12 h without antiparkinsonian medication, using the Hoehn and Yahr scale and the Unified Parkinson's Disease Rating Scale's (UPDRS) subscale III [29]. Patients were also asked to answer the UPDRS subscale II (during “off” state) and the Freezing of Gait questionnaire (FOG-Q) [30]. Higher scores in Hoehn and Yahr, UPDRS-II, UPDRS-III, and FOG-Q correspond to increased severity.

Each PD patient was classified as having tremor-dominant, postural instability and gait difficulty (PIGD), or indeterminate phenotype according to the dominance of the motor symptoms in the UPDRS II and III subscales, following the method described by Jankovic and colleagues [31].

After the neurological examination, a trained psychologist, blinded to the motor assessment scores, applied the DRS-2, the Brief-Smell Identification Test (B-SIT), and the Hospital Anxiety and Depression Scale (HADS). For treated patients (*n* = 161), this nonmotor assessment was conducted under the effect of their regular antiparkinsonian medication (in “on” state).

Both normative sample and PD cohort performed the B-SIT.

#### 2.2.2. Statistical Analyses

Quantile regression models were used to estimate the B-SIT total score for specific percentiles as a function of sex, age, and education. The quadratic effect of age was accounted for in the regression model. Chi-square test, Mann-Whitney test, and multiple logistic regressions were applied to analyze the effects of demographic and clinical variables on PD patients' abnormal B-SIT performance. Quantile regression was fitted with software R v.3.0 and IBM SPSS Statistics 21.0 was used for the remaining statistical analyses. Statistical significance was set at 0.05 for all tests, except for the five direct or indirect indicators of motor severity (i.e., Hoehn and Yahr, UPDRS-II, UPDRS-III, FOG-Q, and levodopa equivalent dose). For the latter, we adjusted the significance level using the Holm-Bonferroni method to take into account the multiple comparisons.

## 3. Results

### 3.1. Normative Data

The B-SIT distribution of the normative sample was leptokurtic (2.5) and negatively skewed (−1.3). Female participants outperformed male participants on the B-SIT (*p* = 0.014). A negative correlation with age (*r* = −0.33; *p* < 0.001) and a positive correlation with education (*r* = 0.28; *p* < 0.001) were found with B-SIT total score. However, the scatter plots suggested that the relation between B-SIT total score and age had a quadratic shape. No significant association (*p* > 0.05) was found with current or past smoking habits.

Algorithms were developed to estimate the B-SIT scores as a function of sex, age, and education for specific percentiles (see Supplementary Material available online at http://dx.doi.org/10.1155/2015/976589). A B-SIT score below the estimated 20th percentile was considered abnormal performance. By definition, this cut-off has a specificity of 80%.

### 3.2. Parkinson's Disease

The prevalence of abnormal olfaction in PD was 82%. Patients with B-SIT scores below the estimated 20th percentile had higher Hoehn and Yahr (*p* = 0.006), UPDRS-III (*p* = 0.018), FOG-Q (*p* = 0.002), and levodopa equivalent dose (*p* = 0.001) than PD patients without abnormal B-SIT scores (Table 1). No significant associations were found with sex, age, education, current or past smoking habits, age at disease onset, disease duration, UPDRS-II, disease subtype, DRS-2 raw scores, and HADS-anxiety and depression scores.

When disease duration was taken into account, the association with Hoehn and Yahr (adj. odds ratio = 4.76; *p* = 0.014), UPDRS-III (adj. odds = 1.06; *p* = 0.032), FOG-Q (adj. odds ratio = 1.24; *p* = 0.009), and levodopa equivalent dose (adj. odds ratio = 1.002; *p* = 0.004) remained statistically significant.

Among PD patients with ≥5 (*n* = 108) or ≥10 (*n* = 41) years of disease duration, the frequency of abnormal olfaction was significantly lower (*p* < 0.05) for those with Hoehn and Yahr stage ≤2.5 than for those with ≥3 (Table 2).

## 4. Discussion

The regression-based norms were used to detect olfactory impairments beyond confounding demographic effects in a cohort with PD. These demographically adjusted B-SIT scores take into account the subtle differences in olfactory function between women and men, the expected decline in smell with age, and the pervasive effect of education in neuropsychological assessment. Demographically abnormal B-SIT scores were present in 82% of nondemented PD patients. The frequency of demographically abnormal olfaction in PD did not vary with sex, age, education, or smoking habits. In other words, olfactory dysfunction due to PD was not influenced by these demographic characteristics. The effects of cognitive functioning in odor identification were not evident in this study, probably because only nondemented PD patients participated in the study.

In this cross-sectional study, abnormal odor identification in nondemented PD patients was associated with more advanced stages of PD (Hoehn and Yahr), with increased severity of motor symptoms (UPDRS-III), including gait disturbance (FOG-Q), and higher dose of antiparkinsonian medication (levodopa equivalent dose), even though disease duration was not a significant predictor.

Multiple logistic regression analysis showed that for a given disease duration the odds of having impaired olfaction increased with disease severity (Hoehn and Yahr, UPDRS-III, FOG-Q, and levodopa equivalent dose). The study results also showed that PD patients with normal olfaction take longer time to reach Hoehn and Yahr ≥3. These results point to a more rapid progression of PD in patients with impaired odor identification and are consistent with Ansari and Johnson's [21] early report of higher thresholds to detect amyl acetate in PD patients with more rapid progression of the disease. Following their seminal work, other studies have reported significant associations between severity of motor symptoms and olfactory functioning in PD, using odor discrimination [23] and odor identification [22, 25, 26] measures. Among these studies with positive results, only Deeb and colleagues [25] explored rate of progression (i.e., symptom severity controlled for disease duration). The authors reported that the University of Pennsylvania Smell Identification Test scores (a larger version of B-SIT) were correlated with UPDRS-III scores even when controlling for disease duration, though this analysis did not differentiate normal from abnormal odor identification.

Motor assessment conditions are an important methodological aspect that has been somewhat neglected by the literature, which may partially explain the variability of findings [2, 5, 12, 14, 17–20, 25]. Unlike most studies on the topic, in our cohort, motor symptoms (i.e., Hoehn and Yahr and UPDRS-III) were consistently evaluated in “off” state (i.e., overnight without antiparkinsonian mediation) to reduce the confounding effect of motor fluctuations. The “off” state evaluation provides a more homogeneous testing condition (between treated and nontreated patients) and a more accurate estimate of disease severity and natural disease progression than the “on” state assessment. The UPDRS-III score is an indicator of global motor condition when applied in “off” state. However, in “on” state, it measures mainly the levodopa resistant symptoms.

Stern and colleagues [22] found that odor identification was less affected in PD patients with tremor-dominant than with predominant PIGD. In our cohort, the frequency of abnormal olfaction tended to be lower among patients with tremor-dominant phenotype (75%) than with PIGD subtype (85%), though the difference was not statistically significant. One possible explanation for this failure to reject the null hypothesis is lack of statistical power. Another possibility is that olfactory functioning and disease subtype are not related. Multiple studies have also failed to reproduce Stern and colleagues' earlier finding [3, 12, 17, 18, 24].

The pathophysiological basis of olfactory dysfunction in PD remains poorly understood. An obvious candidate to explain abnormal olfaction in PD patients is the pathological deposition of *α*-synuclein in primary and secondary olfactory centers [32]. PD-related pathological changes appear early in key sites for olfaction, namely, the anterior olfactory nucleus and the olfactory bulb, and then in closely related areas, such as the olfactory tubercle, the piriform cortex, the periamygdaloid cortex, and the entorhinal cortex. Though neurodegenerative pathology in other anatomical regions may also contribute to odor identification impairment in PD, it has been increasingly recognized that damage to dopaminergic and nondopaminergic neurotransmitter systems may contribute to olfactory dysfunction in PD [33]. SPECT studies have reported robust correlations between nigrostriatal dopaminergic activity and olfactory functioning in PD [15, 16, 26]. The greater reduction in nigrostriatal dopamine transporter binding in patients with abnormal olfaction suggests that these patients have more dopaminergic neuron denervation than those with normal smell. These neuroimaging findings are consistent with our observation that patients with abnormal olfaction have higher disease severity (as measured by motor symptoms and levodopa equivalent dose needs).

## 5. Summary

Olfactory dysfunction due to PD (i.e., beyond demographic confounding factors) is related to disease severity and past disease progression. Its predictive value for future disease progression is unknown and ought to be explored in a long-term prospective study of a well-defined clinical cohort using standardized assessment procedures.

## Supplementary Material

Quantile regression coefficients were used to estimate B-SIT scores for specific percentiles as a function of sex, age, and education. The table presents algorithms to estimate the 5^th^, 10^th^, 15^th^, and 20^th^ percentiles in the normal population.

## Figures and Tables

**Table 1:** Demographic and clinical characteristics of PD patients with and without abnormal olfaction.

	Total sample (*n* = 166)	Abnormal olfaction		*p*
		Yes (*n* = 136)	No (*n* = 30)	
Sex				
Male	87 (52.4%)	70 (80.5%)	17 (19.5%)	0.606
Female	79 (47.6%)	66 (83.5%)	13 (16.5%)	
Age	67 (58–72)	67 (59–73)	66 (57–71)	0.299
Education	4 (4–9)	4 (4–9)	4 (4–10)	0.850
Current smoking habits	8 (4.8%)	6 (75.0%)	2 (25.0%)	0.637
Past smoking habits	38 (22.9%)	32 (84.2%)	6 (15.8%)	0.677
Age at disease onset	59 (50–68)	59 (50–68)	61 (51–62)	0.692
Disease duration	6 (4–9)	6 (4–10)	5 (3–7)	0.105
Hoehn and Yahr	2 (2-3)	2.5 (2-3)	2 (2–2.5)	**0.006**
UPDRS-II	11 (7–16)	11 (7–16)	9 (7–12)	0.052
UPDRS-III	28 (21–34)	28 (22–34)	24 (18–32)	**0.018**
Disease subtype				
Tremor dominant	56 (33.7%)	42 (75.0%)	14 (25.0%)	0.252
PIGD	88 (53.0%)	75 (85.2%)	13 (14.8%)	
Indeterminate	22 (13.3%)	19 (86.4%)	3 (13.6%)	
FOG-Q	3 (1–7)	3 (1–8)	1 (1–4)	**0.002**
Levodopa equivalent dose	640 (400–993)	640 (425–1048)	420 (240–762)	**0.001**
DRS-2	129 (124–133)	129 (124–133)	131 (123–138)	0.384
HADS				
Anxiety	7 (4–9)	7 (4–9)	7 (4–10)	0.803
Depression	6 (4–9)	6 (4–9)	5 (4–8)	0.203

Data are presented as frequencies (%) and medians (25th–75th percentile). Chi-square (or Fisher's exact when appropriate) and Mann-Whitney test were used for group comparisons.

**Table 2:** Frequency of abnormal olfaction in PD patients according to disease duration and disease severity.

	Hoehn and Yahr stage		Chi-square test *p*
	≤2.5	≥3	
Disease duration: ≥5 years	*n* = 69	*n* = 39	0.003
B-SIT <20th percentile	52 (75%)	38 (97%)	

Disease duration: ≥10 years	*n* = 19	*n* = 22	0.049
B-SIT <20th percentile	14 (74%)	21 (96%)	
