# Supplementary material for: Abnormal Olfaction in Parkinson's Disease Is Related to Faster Disease Progression
Source: Behav Neurol. 2015 Jun 2;2015:976589. doi: 10.1155/2015/976589 (PMC4468273; doi:10.1155/2015/976589)
Supplement: Supplementary file 1 — Quantile regression coefficients were used to estimate B-SIT scores for specific percentiles as a function of sex, age, and education. The table presents algorithms to estimate the 5th, 10th, 15th, and 20th percentiles in the normal population. [file 976589.f1.pdf]

Normative Sample data: estimated Brief Smell Identification Test scores for percentiles 5, 10, 15, and 20

Percentiles	Algorithms
5	7.187 – 0.291*sex + 0.083*age – 0.0013*age^2 + 0.055*education
10	6.801 – 0.585*sex + 0.110*age – 0.0015*age^2 + 0.067*education
15	7.408 – 0.919*sex + 0.113*age – 0.0014*age^2 + 0.030*education
20	7.121 – 0.935*sex + 0.123*age – 0.0014*age^2 + 0.038*education

Sex: 0-Women, 1-Men

Sara Cavaco 17/12/14 11:16

Deleted:
